# The effect of the COVID-19 pandemic on immigration and immigrant wellbeing in the United States

**DOI:** 10.1016/j.ssmph.2024.101705

**Published:** 2024-08-12

**Authors:** Sascha Krannich, Douglas S. Massey

**Affiliations:** a Institute for History, Theory and Ethics of Medicine, Giessen University, Leihgesterner Weg 52, 35392, Gießen, Germany; b Sociology and Public Affairs, Princeton University, 239 Wallace Hall, 08544, Princeton, NJ, United States

**Keywords:** Health, COVID-19, United States, Immigration, Immigrant wellbeing

## Abstract

This article highlights the effect of the COVID-19 pandemic on immigration.

and immigrant wellbeing in the United States by focusing on all categories of migrants, documented and undocumented. We argue that in the wake of the pandemic, immigrants disproportionately experienced higher rates of unemployment, greater losses of income, more exposure occupational risks, and higher rates of food and housing insecurity, all of which exacerbated preexisting differentials in access to health and health care to generate higher rates of COVID infection, morbidity, and mortality among adults and stunted educational outcomes for their children. The prospects for a full post-pandemic recovery of immigrants' wellbeing are dampened by the severe nature of COVID's negative effects on immigrants; the unusually hostile context of reception immigrants face after the pandemic; the large number of immigrants lacking legal status or holding tenuous documentation; and the formidable deportation regime that prevails in the United States that puts a great strain on immigrant communities. Undocumented migration has surged to restart undocumented population growth, further clouding the future for immigrants in the country. It is unclear whether reforms proposed by the Biden Administration be enacted and successful in improving their prospects. In general, this article aims to contribute to the broader discussion about migration and health policies.

## Highlights

• Immigrants disproportionately experienced higher rates of unemployment, greater losses of income, and more exposure occupational risks.
• Rising anti-immigrant prejudice and discrimination, with animus focused particularly on refugees, asylum seekers, the undocumented, and minority group members.
• Policy makers need to address the social and economic effects of the COVID-19 pandemic and migrant wellbeing with realistic plans to accommodate the flows.
• Improvement and assurance of all migrants (including refugees and undocumented) to health and health care.

## 1 Introduction

More than three years after the COVID-19 pandemic began in China (sometime in November 2019), officials declared an end to the public health emergency in the United States on May 11, 2023. Over the preceding years, the pandemic seriously disrupted international migration to the United States, cutting the annual number of immigrants, curtailing their access to health care, education, and housing, and sparking a rise in anti-immigrant prejudice and discrimination, with animus focused particularly on refugees, asylum seekers, the undocumented, and minority group members (see Clark et al., 2020; Bhuiyan et al., 2021; Mula et al., 2022; Page et al., 2020; Tai et al., 2021; Van et al., 2021). Here we examine the effects of COVID-19 on immigration and immigrant wellbeing in the United States and consider possible future trajectories with respect to both outcomes.

## 2 COVID-19 impact on immigration flow

In response to the pandemic, countries worldwide closed their borders, enacted travel bans, and paused visa processing, dramatically curtailing the cross-border movement of migrants. U.S. consulates and embassies stopped issuing visas in March of 2020, and in April the Trump Administration banned labor and family immigration, creating havoc for permanent and temporary migrants alike (Gelatt & Chishti, 2022; Passel and D'Vera, 2022).

### 2.1 Impact across Different Immigrant groups

Between 2019 and 2020, entries into the United States by legal immigrants dropped by 31% overall, and admissions by relatives of U.S. citizens and legal residents declined by 38%, preventing or at least delaying many family reunifications (U.S. Office of Immigration Statistics, 2022). Although entries by workers on permanent visas rose by 7% between 2019 and 2020, the allocation of temporary work visas ceased in June of 2020. Exceptions to the cutoff were issued for “necessary” H-2A agricultural workers and some H-2B service workers (Congressional Research Service, 2020). Nonetheless, in total temporary worker entries fell by 37% between 2019 and 2020, leaving many workers unable to reach the jobs to which they had been recruited (U.S. Office of Immigration Statistics, 2022).

Persons entering the United States on temporary visas are not considered to be immigrants, and across all nonimmigrant admission categories entries fell by 54%. Entries on student and exchange visas dropped by 54%, blocking many students and scholars from reaching academic institutions where they had been admitted. Others were prevented from returning to campus after going home for the holidays. At the same time, many vacations and business trips were aborted, as tourist entries dropped by 56% and business entries fell by 54% (U.S. Office of Immigration Statistics, 2022).

### 2.2 Refugees and asylum seekers

The year 2020 thus proved to be difficult for international migrants, who faced great uncertainty about their prospects for being able to stay, enter, or leave countries of origin, transit, and destination. Circumstances were perhaps hardest for refugees and asylum seekers, as they were presumably fleeing serious threats to their wellbeing at places of origin Lerpold et al., 2023). Both the United Nations High Commissioner for Refugees (UNHCR) and the International Organization for Migration (IOM) suspended resettlement efforts from March to July 2020, leaving many migrants stranded enroute without access to adequate healthcare, sanitation, or lodging. Between 2019 and 2020, the global population of refugees rose by 1.2% in 2020 (UNHCR 2023) as overcrowding in refugee camps increased, food scarcities arose, and migrant health deteriorated via exposure extremes of heat and cold and inadequate shelter (United Nations, 2020, p. 8).

Although some European countries continued to admit refugees and asylum seekers during the pandemic, in the United States the Trump Administration invoked Title 42 of the Public Health Service Act on March 20 of 2020. This action sought to block migrants from seeking affirmative asylum at ports of entry, and to expel those who sought defensive asylum after undertaking an unauthorized entry ( Centers for Disease Control and Prevention, 2023). A few months later, at the end of September, Trump cut the target quota for refugee admissions to the lowest level in recorded history (Miroff, 2020).

Although these policy shifts were ostensibly implemented to protect the health of U.S. residents, most observers saw it as a continuation of Trump's pre-pandemic efforts to reduce immigration, and they certainly had that effect. Between 2019 and 2020, the number of refugees granted permanent residence dropped 45% and asylees admitted to permanent residence fell by 25% (U.S. Office of Immigration Statistics, 2022).

These actions occurred despite a rising tide of forced migration throughout the world, owing not only to the pandemic and the economic dislocations it brought, but also to the cumulative effects of climate change, rising authoritarianism, growing criminal and civil violence, and institutional failures (Arar & FitzGerald, 2023; Massey, 2023). According to the UNHCR (2023), the global population of refugees grew by another 3.2% between 2020 and 2021 and the global population of asylum seekers rose by 10.3%. In contrast, the number of refugees admitted to the United States fell by 3.3% and asylum approvals plummeted by 42.9% (U.S. Office of Immigration Statistics, 2022).

### 2.3 Border enforcement

Trump's efforts to cut refugee and asylum admissions during a global humanitarian crisis were accompanied by a sharp increase in the enforcement efforts along the nation's borders and within its interior. According to the U.S. Office of Immigration Statistics (2022), immigrant apprehensions tripled between 2020 and 2021 and the number deportations rose by a factor of five. Over the same period, the number of migrants declared inadmissible rose by 22% and the number of expulsions under Title 42 increased 5.2 times (U.S. Office of Immigration Statistics, 2022).

**Fig. 1:**
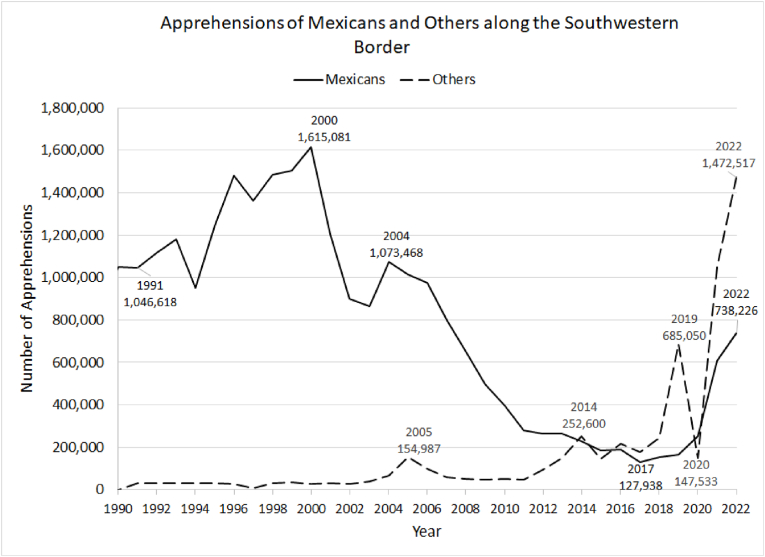
Apprehensions of Mexicans and others along the Southwestern border.

Source: U.S. Customs and Border Protection, 2023.

The overwhelming majority of apprehensions and expulsions were carried out by U.S. Border Patrol agents operating at or near the Mexico-U.S. border, and the global shift from workers and family members to forced migrants and dependents is readily apparent in the number and composition of those apprehended (Massey, 2020a; 2020b). Fig. 1 shows that the number of non-Mexicans apprehended dropped to a low of 148,000 during the pandemic year of 2020 from a peak of 685,000 in 2019, then rose once again to a crest of nearly 1.5 million in 2022, bringing total apprehensions to an unprecedented 2.2 million. The number of Mexican apprehensions rose to 738,000 in 2022, not itself a record but still quite large (U.S. Customs and Border Protection, 2023).

In the revival of unauthorized migration that occurred as the pandemic wound down during 2022, we see that single adults clearly dominated among Mexicans whereas family units and single minors dominated among those apprehended from the Northern Triangle and other regions. Whereas 93% of Mexicans apprehended in 2022 were single adults, the figure was just 24% for Northern Triangle migrants and 35% for migrants originating in other nations. Instead, the former set of migrants consisted 21.9% of single minors and 54.1% of migrants in family units, and the latter comprised 63.7% traveling in family units and 1% moving as single minors (U.S. Customs and Border Protection, 2023).

These shifts in composition might be taken to indicate that the post-pandemic revival of unauthorized migration from Mexico return to large-scale labor migration whereas unauthorized migration from the Northern Triangle and elsewhere in Latin America consisted primarily of forced migrants seeking refuge. The picture for Mexico is clouded, however, by the fact that in the years leading up to the pandemic, Mexican crossing the border for work were increasingly legal temporary laborers as opposed to unauthorized migrants (see Wassink & Massey, 2022).

Instead, outflows of migrants from Mexico seem increasingly to be comprised of adults fleeing weather events associated with global climate change (Riosmena et al., 2018; Molina et al., 2022; Thalheimer et al., 2022), movers seeking to escape Mexico's rising tide of lethal violence (Basu & Pearlman, 2017; Massey et al., 2020), and those displaced from jobs owing to pandemic-related economic disruptions. These trends are in keeping with the broader global shift away from migrants who voluntarily move to access opportunities and toward those moving to escape threats to wellbeing (Massey, 2023). Of course, they could also be workers seeking U.S. employment after being displaced from jobs owing to pandemic-related economic disruptions (Wassink and Massey. 2022).

## 3 Barriers to health and health care

Although the laws and regulations that determine migrants' access to health care and health insurance differ from state to state, fundamental barriers to immigrants' access to health care nonetheless prevail throughout the nation (Castañeda et al., 2015; Clark et al., 2020). Already before the pandemic, structural factors substantially blocked immigrants’ access to the U.S. medical system (Pirtle, 2020). The obstacles to receiving care include poor insurance coverage, limited financial resources, legal and bureaucratic prohibitions, limited knowledge of the U.S. health system, long waiting times for services, and language barriers, as well as rising exposure to nativist prejudice and discrimination (Hacker et al., 2015; Derr, 2016; Đoàn et al., 2021).

### 3.1 Documented immigrants

Documented immigrants are dissuaded from seeking health care because of the public charge rule, a legal prohibition that threatens to deny or strip U.S. permanent residence from legal immigrants who receive public assistance (Castañeda & Melo, 2014). The rule was revised to be considerably more restrictive by the Trump administration in February 2020 (Đoàn et al., 2021). Although the public charge rule was withdrawn by the Biden administration in March of 2021, fears and confusion about using public assistance persist in immigrant communities (Beier & Workie, 2022). At the beginning of the pandemic, the US government expanded access to public benefits in the frame of the 2010 Affordable Care Act. That helped particularly low-income migrant families, but was restricted to documented immigrants and excluded undocumented immigrants. The expanded access to public benefits ended with the official end of the pandemic in May 2023 (Lacarte et al., 2024).

### 3.2 Undocumented immigrants

Undocumented migrants face by far the greatest barriers to accessing health care in the United States (Fleming et al., 2019; Wallace & Young, 2018). In general, they are prohibited from receiving all but emergency services and are excluded from federal health benefits and insurance programs under both the 1996 Personal Responsibility and Work Opportunity Reconciliation Act and the 2010 Affordable Care Act (Hacker et al., 2015; Wallace et al., 2013). Moreover, although the Coronavirus Aid, Relief, and Economic Security Act (CARES Act) and the Families First Coronavirus Response Act (FFCRA) passed in March of 2020 offered free COVID-19 testing for the uninsured, neither legislative packaged funded treatment for those found to be infected.

In addition, the Recovery Rebate Credits authorized by the CARES Act were sent only to persons with a valid Social Security number, excluding many immigrants who use false numbers or Individual Taxpayer Identification Numbers to file tax returns (Hill et al., 2021; National Immigration Law Center, 2022). Even before the pandemic, undocumented migrants were reluctant to seek medical care for fear of creating a documentary record that could alert authorities to their presence in the country, leading to possible detention and deportation. Undocumented migrants consequently tend to delay needed treatment until the threat to health is so severe that they end up in emergency wards (DeSalvo et al., 2016). Undocumented parents are also less likely than others to treat health issues experienced by their U.S.-born citizen children (Gelatt, 2016; Vargas and Vickie, 2017); and after vaccines became available in early 2021, undocumented migrants were reluctant to get them for themselves and even their children, presenting a clear challenge to public health authorities in immigrant-receiving areas (Matlin et al., 2022).

In short, the anti-immigrant policies implemented under President Trump functioned to compound and intensify the negative health effects of the pandemic for undocumented migrants (Garcini et al., 2020). The threats to health and wellbeing are especially multiplied for those undocumented migrants who are arrested and detained, a risk that is by no means trivial (seeAsad and Matthew, 2018). During the first half of 2023, for example, the U.S. government reported a total of 1.2 million expulsions, removals, and returns (U.S. Department of Homeland Security 2021). As of June 2023, the average daily count of immigrants in detention was around 30,000, which corresponds to an estimated annual detainee population of around 358,000 persons (TRAC Immigration 2023). Deportations and detentions increased the spread of the virus (Casanova et al., 2021). From February 2020 to January 2021, some 9099 migrants tested positive for COVID while in detention (Đoàn et al., 2021), and infections spread rapidly among detainees because of unsanitary living conditions, overcrowding, and limited access to medical care while in custody (Okonkwo et al., 2020).

In sum, the pandemic exacerbated these preexisting circumstances to expand inequities in health and access to medical care between immigrant and native communities, with deadly results (Clark et al., 2020; Rodriguez et al., 2021). COVID-19 morbidity and mortality rates were significantly higher among immigrants and minority groups compared to native and White Americans (Hasan Bhuiyan et al., 2021). In the first year of the pandemic, rates of infection and mortality among Hispanics (a third of whom are foreign born) were much higher than among Whites (COVID Tracking Project 2023).

## 4 Loss of employment and income

In all Western Nations, immigrants work in jobs that are essential to keep economies running and societies functioning (see OECD 2022). In the United States, immigrants account for 17% of the entire labor force, but they are overrepresented in several labor-intensive professions such as agriculture (where they comprise almost half the workforce) and the food industry (almost 40% of the workforce). They are also overrepresented in diverse other sectors such as sales, distribution, hospitality, public transport, and health care. In the latter industry, 29% of all physicians and 22 % of nurses are immigrants (Gelatt & Chishti, 2022, p. 11). Without their crucial labor, the societal impact of the pandemic would likely have been even worse.

### 4.1 Bad working conditions

In occupations where they concentrate, however, immigrants are often poorly paid, work limited hours, and are likely uninsured. In addition to being dangerous, demeaning, and unhealthy, immigrant jobs tend to be unstable and ununionized, leaving them vulnerable to economic downturns. Although immigrant unemployment rates vary from state to state (Capps et al., 2021), from the beginning of the pandemic through the second quarter of 2020 their average rate rose from 4.1% to 15.3% compared to an increase of 4.0%–12.4% for native workers (US Bureau of Labor Statistics 2021).

Among Latin American immigrants the rate rose even higher, reaching an average level (16.2%), which exceeds even the maximum displayed by immigrants during the Great Recession of 2008 (Kochhar & Jesse, 2020). As always low-income immigrants were particularly vulnerable, and in one nationally representative survey, 26% reported that either they or their partner had experienced a job loss, with the same percentage stating that a family member had been furloughed or given reduced work hours leading to reduced income (Bernstein et al., 2021). Job losses were especially acute for undocumented migrants (Borjas and Hugh, 2020). These high rates notwithstanding, as already noted immigrants do not enjoy the same social safety net protections as natives owing to legal ineligibility, fears of violating the public charge rule, language barriers, and widespread confusion about how to apply for benefits (Amandolare et al., 2020).

Although employment rates recovered during the economic upswing of 2021, immigrants continued to work in poorly paid, insecure, and unsafe jobs, especially women (U.S. Bureau of Labor Statistics, 2022; Kochhar and Bennett 2022). In these jobs, migrants typically labored alongside densely packed coworkers, exposing them to a greater risk of COVID-19 infection, most notably in agriculture (Handal et al., 2020) and food processing (Smith, 2000; Waltenburg et al., 2020).

In April of 2020, for example, the Smithfield pork processing plant in South Dakota became the nation's number one hotspot with more than 1000 COVID-19 cases (Corkery and Yaffe-Bellany 2020). A study of meat processing facilities in Nebraska showed an infection rate of 19% among workers between April and July 2020 (Herstein et al., 2021). In October of 2021, a Congressional investigation confirmed high numbers of COVID-19 incidences all over the nation (Gelatt & Chishti, 2022). Of course, outbreaks in food processing facilities led to the spread of the virus into surrounding communities ( US Department of Agriculture, 2021).

## 5 Living conditions and education

### 5.1 Deteriorating housing

In addition to the COVID-related risks they face owing to the kinds of jobs they hold, immigrants are also at greater risk owing to the households they occupy, the housing they inhabit, and the neighborhoods in which they reside. For example, immigrants are more likely than natives to reside in multigenerational families (Fry & Passel, 2014), and so have an elevated risk of COVID transmission, because such households are larger, are more likely to include older adult members, and often contain essential workers (Đoàn et al., 2021).

Owing to residential segregation, immigrants are also likely to occupy poor quality housing in more disadvantaged neighborhoods characterized by a limited availability of health and social services, including medical care (Riley, 2018). Under these circumstances, they are also subject to excessive rent burdens and elevated risks of eviction (Hernández et al., 2016). Immigrant neighborhoods are also characterized by a paucity of grocery stores with fresh and healthy food, increasing the prevalence of food scarcity and hunger, which were exacerbated by systematic disenrollment immigrants from entitlements such as the federal Supplemental Nutrition Assistance Program under President Trump (Lê-Scherban et al., 2023; Page et al., 2020).

### 5.2 Schools and learning

Because students in the United States are allocated to public schools largely based on the districts and neighborhoods that they and their families inhabit, residential segregation is strongly correlated with school segregation (Massey and Jonathan, 2016). Owing to this structural connection, the disadvantaged neighborhood circumstances of immigrants translate directly into in disadvantaged schools for the children of immigrants (Owens, 2017; Rich & Owens, 2023). It is not surprising, therefore, that in its review of research on the consequences of COVID, the National Academies of Science, Engineering, and Medicine (2023) documented declines for immigrant-origin children across a range of school engagement and learning outcomes over the course of the pandemic.

The shift to remote learning during the spring of 2020 and the ensuing academic year was particularly difficult for the children of immigrants, many of whom lacked access to reliable internet services and personal computers. They also lived in more crowded households, creating distractions from studying; and often their parents had limited English proficiency, making it difficult for them to follow instructions from the schools and to provide support for online assignments (Greenberg, 2021).

At the same time, both parental employment and unemployment paradoxically create problems for immigrant households (Oliveira & Segel, 2022). One the one hand, if a parent remained employed despite the economic shutdown, their absence from the home during working hours led to shortfalls to the care and supervision of children furloughed from schools. Parents who remained in the labor force also often worked in “essential” frontline occupations that increased their risk of infection and thus raised the risk of transmitting COVID to their children. On the other hand, parents who became unemployed during the shutdown experienced losses of income that put their households at economic risk during a time when investments were required to achieve online and access medical care ( National Academies of Science, Engineering, and Medicine, 2023).

Finally, interactions with the immigration bureaucracy and especially its enforcement apparatus created additional strains that exacerbated the normal pressures and risks of the pandemic, especially whenever parents were detained (Oliveira & Segel, 2022). Đoàn et al. (2021: 226–227) sum up the situation facing immigrant families by noting that “the cumulative financial strain from unemployment, income loss, and housing insecurity complicates access to health care services and increases risk of household transmission of COVID-19 for immigrant communities.”

## 6 Increased prejudice and discrimination

The COVID-19 pandemic and its ensuing health insecurities led also to rising prejudice and discrimination against migrants and other minority groups who were accused of being responsible for the outbreak and spreading of the pandemic. Racist and xenophobic sentiment was fueled by leading American politicians, most notably President Donald Trump, who called COVID-19 the “Chinese Virus” and blamed China and Chinese immigrants for spreading the virus into the United States (Rogers et al., 2021). Other prejudicial terms bandied about the public sphere were the labeling of COVID as the “Wuhan Virus” and the “Kung Flu” (Walker & Anders, 2022).

In this political context, it is hardly surprising that anti-Asian discrimination rose in the wake of the COVID-19 pandemic. A nationwide survey done by the Pew Research Center in June of 2020 found that among Asians, 39% perceived that after the outbreak other people were more uncomfortable around them because of their race. After the COVID outbreak began, 31% of Asians reported being subject to racial slurs or jokes, and 26% stated they feared someone might threaten or attack them, whereas the respective percentages for Whites were just 13%, 8%, and 11% (Ruiz et al. 2020). In the same survey, 36% of Asians said they worried that others would be suspicious of them if they wear a mask, compared to just 5% of Whites.

The number of anti-Asian hate crimes reported to police in America's 16 largest cities rose 149% between 2019 and 2020 even though overall hate crimes dropped by 7% ( Center for the Study of Hate and Extremism, 2020). Ruiz et al. (2020), found that 58% of Asians believed it had become more common for people to express racially insensitive views about them than it was before the pandemic, whereas only 18% of Whites expressed this view about their group. Another nationally representative survey of 5500 adults fielded from December 2020 to February 2021 found that 30% of Asians had experienced some form of anti-Asian discrimination since the pandemic's onset whereas only 10.4% of Whites perceived and increase in anti-White discrimination (Strassle et al., 2021). In the same study, after the pandemic's onset 44.1% of Asians perceived people to act afraid in their presence, compared to 33.4% of Whites.

An analysis of 9081 hate incidents reported to the organization “Stop AAPI Hate” from March 19, 2020 to June 30, 2021 found that 63.7% of the incidents entailed verbal harassment, 16.8% involved avoidance or shunning, 13.7% entailed a physical assault, and 8.5% involved being coughed or spat upon ( Yellow Horse et al. 2021). Among the reported incidents, 31.6% occurred on a public street or sidewalk, 30.1% transpired at a place of business, 9.4% at a private residence, 8.8% online, 8.5% on public transit, 8.1% in a public park, and 6.0% at school. Among incidents that involved hateful language, 21.7% scapegoated China, 17.8% were racial slurs, 13.2% were expressions of nativist sentiment, and 8.1% involved orientalist caricatures (Yellow Horse et al. 2021).

Although Asians in general and Chinese in particular may have been singled out for attack in the wake of the COVID outbreak, they were by no means alone. Ruiz et al. (2020) found that 38% of Blacks reported people being uncomfortable around them since the COVID outbreak, just one point below the percentage for Asians. Strassle et al. (2021) likewise reported that 26.9% of Latinos and 20.5% of Blacks had experienced some form of discrimination against their group since the pandemic began, both well above the White share of 10.4%. Among Latinos, experiences of discrimination were far more frequent among those interviewed in Spanish (36.1%) than among those interviewed in English (17.5%), suggesting that prejudice is greater for Hispanics suspected to be foreign born, undocumented, of more culturally Latino. Unfortunately, this finding could not be replicated for Asians, who were only interviewed in English.

A population-based sample of Chinese American families with children aged 10–18 surveyed in the United States from March 14, 2020, to May 31, 2020, found that 76.8% of parents and 76.5% of their children reported at least one incident of COVID-related racial discrimination online and/or in person (Cheah et al., 2020). In addition, some 49.1% of parents and 71.1% of children youth perceived health related Sinophobia in America, whereas 50.4% of parents and 56.0% of children perceived Sinophobia being perpetuated in the media, and that perceptions of racism and racial discrimination were associated with their poorer mental health.

Studies reveal that experiences and perceptions of discrimination have a significant negative influence on mental health. A cross-national study of immigrants from 30 countries found that perceived discrimination strongly predicts symptoms of depression, anxiety, and paranoia among immigrants throughout the world (Brance et al., 2022). A survey of Chinese immigrants in North Carolina similarly concluded that “discrimination aggravates the psychological burden of multiple stressors in Chinese immigrants' lives by uniquely contributing to perceptions of stress alongside contemporaneous stressors” (Stolte et al., 2022).

## 7 Conclusion and discussion

In the wake of the pandemic, evidence indicates that immigrants disproportionately experienced negative outcomes compared to natives, with higher rates of unemployment, greater losses of income, more exposure occupational risks, and higher rates of food and housing insecurity, all of which exacerbated preexisting differentials in access to health and health care to generate higher rates of COVID infection, morbidity, and mortality among adults and stunted educational outcomes for their children.

It is too early to project with any confidence what the long-term consequences of these setbacks might be. Although the wellbeing of immigrants has gradually improved since the pandemic ended, the best they can probably hope for is a return to their disadvantaged pre-pandemic status in the U.S. stratification system (National Academies of Science, Engineering, and Medicine, 2023). But even that modest receiver is not assured, for the reasons adumbrated below.

First is the sheer severity of COVID's deleterious effects on immigrants and their children compared to natives. Among adults, the excess in lost jobs, lost hours of work, and lost earnings can never be recovered, permanently reducing lifetime incomes and degrading accumulations of human, social, and financial capital. In addition, the disproportionate losses among adult immigrants, combined with school closures and the switch to remote learning in 2020, put their children even further behind educationally relative to their native peers, creating learning deficits that are hard to make up.

In addition, since the pandemic's ending the children of immigrants have not returned to school at the same pace as natives. Some were compelled by family circumstances to enter the labor force and others tried to return to school but left because they had fallen too far behind to catch up. More ominously, with the upturn in hostility toward immigrants many parents became wary of sending their children back to school, and the children themselves were often fearful of returning (Gándara et al., 2023). In sum, the context of reception for immigrants noticeably deteriorated during the pandemic. Therefore, it needs more policy efforts to address disadvantaged immigrant employees and their children, including more workplace protections (particularly providing adequate breathing equipment and quarantine rooms in sectors where vulnerable workers work under health risky conditions), homeschooling facilities as well as access to after-school care. Universal relief programs, including food assistance, pandemic stimulus payments, and expanded child tax credit, which were adopted by the U.S. government, and which also helped struggling immigrant families, went into the right direction.

According to Portes and Rumbaut (2014), the context of reception is the constellation of social, economic, and legal circumstances that determine the degree to which immigrants are accepted by natives and thus able to integrate freely within a host society. The deterioration of immigrants' context of reception over the course of the pandemic constitutes a second reason for offering a guarded assessment of immigrants’ mobility prospects moving forward.

Reactionary nativism and xenophobia were on the rise well before the advent of COVID-19, of course (see Parker and Barreto 2013; Abrajano and Hajnal 2017). But increased nativism and overt racism both contributed to and were intensified by the election of Donald Trump (Hajnal 2021; Parker 2021; Hout and Maggio 2021; Canizales and Vallejo 2021). His electoral success gave him a popular mandate to impose new limits on documented as well as undocumented migration to the United States (Verea 2018) and fresh license to double down on his strident anti-immigrant rhetoric (Sagir and Mockabe 2022).

What Chavez (2013) identified as an “Anti-Latino Threat Narrative” had been building in the U.S. media since the mid-1980s when the United States began to militarize the Mexico-U.S. frontier (Chavez 2013). Border militarization produced more apprehensions, which served to confirm the existence of the continuing “invasion” by “illegal aliens,” justifying even more restrictive enforcement actions, which in turn produced still more apprehensions, ultimately turning border militarization into a self-perpetuating process (Massey und Pren 2012).

Therefore, a decisive approach against racism and discrimination is necessary (not only regarding Latinos, but of all immigrant groups). Besides reactive measures, there are also preventive measures mandatory, such as racial awareness and critical education, not only for students in the frame of their curricula, but also for employees in companies, organizations, and state institutions.

Given the social and economic effects of the pandemic, global climate change, and civil violence prevailing in the southern portion of the Western Hemisphere, additional population movements toward the Mexico-U.S. border are a foregone conclusion. What is needed are policies that accept this reality with realistic plans to accommodate the flows. Decades of border militarization and 6.5 million deportations since 2000 have not solved the problem but have exacerbated existing and created new humanitarian crises (see Massey and Jonathan, 2016; Massey, 2020a, 2020b; Solano & Massey, 2022). Instead, policies should enable more channels for legal short- and long-term immigration for educational, working, and family purposes. In a health crisis, travel and immigration restrictions should be only for the required time to protect the lives of immigrants as well as citizens. They should be removed immediately when they are not needed anymore, accompanied by sufficient quarantine facilities and access to health care, as it was the case in some European countries, where the COVID effects on migrants were not as harsh as in the United States (Thränhardt 2023). However, undocumented migrants and refugees suffered the most under COVID-related restrictions worldwide.

Joseph Biden was elected President on a promise to adopt more humane and efficacious immigration and border policies, and in March of 2023, he announced a proposed budget package “to secure our border and rebuild a safe, orderly, and humane immigration system that was gutted by the previous Administration” (White House 2023). In deference to political reality, the package necessarily leads with $800 million in additional funding for border and interior enforcement, but it goes on to open additional pathways for legal entry into the United States.

First, the package budgets $865 million to handle the rising asylum caseloads and process the admission of up to 125,000 refugees per year. Second, it creates a $4.7 billion contingency fund to respond to future migration surges along the border and allocates more than $1.5 billion to reduce the backlog of over 1.8 million cases currently pending in the immigration court system. Third, it provides $7.3 billion to the Office of Refugee Resettlement (ORR) to rebuild the nation's refugee resettlement infrastructure, which was scuttled under President Trump.

Finally, Biden's package budgets $291 million to strengthen Haiti's National Police, allocates another $430 million for hemispheric migration management, and invests $50 million to create a new regional economic opportunity fund. In addition to these budgetary initiatives, upon assuming office he began to reign in the nation's deportation machine, reducing the number of removals from 238,000 in 2020 to just 89,000 in 2021, the lowest number since 1996. Then in January of 2023, he announced that effective immediately he would use the parole authority granted him under the 1952 Immigration and Nationality Act to admit up to 30,000 nationals from Cuba, Haiti, Nicaragua, and Venezuela into the United States each month; and in May of 2023 he ended Trump's policy of Title 42 expulsions along the border.

It remains to be seen whether these initiatives will be fully enacted in the face of Republican opposition and, if enacted whether they will be successful in checking the renewed growth in the size of the nation's undocumented population, but at least they are a step in the right direction. Moving forward the nation needs not only to enact measures to counter growth in this population; it also needs to find a way to legalize the millions of long-term undocumented residents who have peaceably integrated north of the border. The condition of mass illegality owing to a lack of documentation among Hispanics, now the nation's largest minority group, is unsustainable and a fundamental threat to the creation of a fairer and more egalitarian society and a functioning health system.

## Ethical statement

We assure that this research did not collect data from human subjects.

## CRediT authorship contribution statement

**Sascha Krannich:** Writing – review & editing, Writing – original draft, Investigation, Formal analysis, Data curation, Conceptualization. **Douglas S. Massey:** Writing – review & editing, Writing – original draft, Methodology, Investigation, Funding acquisition, Formal analysis, Data curation, Conceptualization.

## Declaration of competing interest

The authors declare that they have no known competing financial interests or personal relationships that could have appeared to influence the work reported in this paper.

## Data Availability

Data will be made available on request.
